# Melatonin enhances antioxidant molecules in the placenta, reduces secretion of soluble fms-like tyrosine kinase 1 (sFLT) from primary trophoblast but does not rescue endothelial dysfunction: An evaluation of its potential to treat preeclampsia

**DOI:** 10.1371/journal.pone.0187082

**Published:** 2018-04-11

**Authors:** Natalie J. Hannan, Natalie K. Binder, Sally Beard, Tuong-Vi Nguyen, Tu’uhevaha J. Kaitu’u-Lino, Stephen Tong

**Affiliations:** Translational Obstetrics Group, Mercy Perinatal, Department of Obstetrics and Gynaecology, University of Melbourne, Mercy Hospital for Women, Heidelberg, Victoria, Australia; University Hospital Basel, SWITZERLAND

## Abstract

Preeclampsia is one of the most serious complications of pregnancy. Currently there are no medical treatments. Given placental oxidative stress may be an early trigger in the pathogenesis of preeclampsia, therapies that enhance antioxidant pathways have been proposed as treatments. Melatonin is a direct free-radical scavenger and indirect antioxidant. We performed *in vitro* assays to assess whether melatonin 1) enhances the antioxidant response element genes (heme-oxygenase 1, (HO-1), glutamate-cysteine ligase (GCLC), NAD(P)H:quinone acceptor oxidoreductase 1 (NQO1), thioredoxin (TXN)) or 2) alters secretion of the anti-angiogenic factors soluble fms-like tyrosine kinase-1 (sFLT) or soluble endoglin (sENG) from human primary trophoblasts, placental explants and human umbilical vein endothelial cells (HUVECs) and 3) can rescue TNF-α induced endothelial dysfunction. In primary trophoblast melatonin treatment increased expression of the antioxidant enzyme TXN. Expression of TXN, GCLC and NQO1 was upregulated in placental tissue with melatonin treatment. HUVECs treated with melatonin showed an increase in both TXN and GCLC. Melatonin did not increase HO-1 expression in any of the tissues examined. Melatonin reduced sFLT secretion from primary trophoblasts, but had no effect on sFLT or sENG secretion from placental explants or HUVECs. Melatonin did not rescue TNF-α induced VCAM-1 and ET-1 expression in endothelial cells. Our findings suggest that melatonin induces antioxidant pathways in placenta and endothelial cells. Furthermore, it may have effects in reducing sFLT secretion from trophoblast, but does not reduce endothelial dysfunction. Given it is likely to be safe in pregnancy, it may have potential as a therapeutic agent to treat or prevent preeclampsia.

## Introduction

Complicating around 3% - 5% of all pregnancies, preeclampsia is a leading cause of maternal mortality, especially in developing countries [1, 2]. It is estimated to be responsible for over 60,000 maternal deaths annually [3] and far higher rates of perinatal loss [3]. Since there are no treatments to arrest disease progression, the only known way to stop this disease is delivery of the placenta, removing the source of the pathogenic circulating factors that contributes to the maternal disease. As such, preeclampsia is a major cause of premature delivery, especially when preeclampsia occurs at preterm gestation (<34 weeks). Thus, an effective therapy to treat or prevent preeclampsia would be a major advance, saving many lives globally.

Preeclampsia is a multi-system disorder affecting maternal vessels (causing high blood pressure (hypertension) and vascular injury (endothelial dysfunction)), kidneys, liver, the haematological system, brain (causing seizures or eclampsia) and the fetoplacental unit (resulting in fetal growth restriction) [3]. In preeclampsia, hypertension is characterized by the mother’s peripheral vasoconstriction and decreased arterial compliance [4] and the high levels of protein in the urine comes from acute inflammation of the kidneys endothelial cells (lining the glomerular) [4], which leads to significant tissue damage and poor filtration.

Placental and systemic oxidative stress is thought to be a key aspect in the pathogenesis of preeclampsia [5]. It is widely thought that the pathological process of preeclampsia can be attributed to two main events: inadequate implantation and subsequent maternal endothelial dysfunction [3]. In preeclamptic pregnancies, the placenta fails to adequately invade and remodel the maternal uterine spiral arteries. This compromises placental perfusion, resulting in high levels of oxidative stress in the placenta [5–7]. The dysfunctional stressed placenta releases excess levels of anti-angiogenic factors, specifically soluble fms-like tyrosine kinase 1 (sFLT) [8, 9] and soluble endoglin (sENG) [10, 11] into the maternal circulation, where they injure the maternal vasculature causing widespread endothelial dysfunction and multi-system maternal organ injury [3, 4, 12]. A treatment that can reduce the oxidative stress in the preeclamptic placenta could help to reduce tissue damage. Such a therapy could be a novel strategy to prevent the disease, or decrease severity.

Antioxidants are molecules that inhibit the oxidation of other molecules, and thus reduce or stop the chemical chain reaction of free radical production that results in oxidative stress [13]. Melatonin (5-methoxy-N-acetyltryptamine), a potent endogenous antioxidant, functions both directly as a free radical scavenger and indirectly by activating antioxidant enzymes [14–16]. Melatonin is primarily synthesized in the pineal gland [17], however throughout gestation the placenta is a significant extrapineal source, with maternal melatonin levels peaking at term [18, 19]. As well as synthesis, melatonin readily crosses the placental barrier and acts to promote trophoblast survival through its MT1 and MT2 receptors [19–21]. Aside from cellular antioxidant defenses, melatonin also has vasodilatory properties [22–24]. Melatonin is largely considered vital in pregnancy success [25].

Circulating melatonin levels are significantly decreased in cases of severe preeclampsia [26] concurrent with decreased placental melatonin synthesis and receptor abundance [27]. Serotonin, melatonin’s immediate precursor, is increased in preeclamptic placentas due to an inhibition of aralkylamine N-acetyltransferase activity, the rate limiting enzyme in melatonin synthesis [27]. Use of melatonin as a therapeutic in animal models of placental ischemia has shown reduced tissue and DNA damage from oxidative stress [28, 29], with no reported negative side-effects [30]. Given this, and the likely safety of melatonin supplementation during pregnancy for mother and fetus, we set out to test the possibility that melatonin may be a treatment for preeclampsia in models of preeclampsia in vitro.

## Materials and methods

### Tissue collection

Ethical approval was obtained for this study from the Mercy Health Human Research Ethics Committee. Women presenting to the Mercy Hospital for Women, Melbourne gave informed written consent for tissue collection.

Placentas and umbilical cords were obtained from normal term pregnancies (>38 weeks gestation) at elective cesarean section for functional studies. Placentas and umbilical cords were collected within 30min of delivery and washed in sterile phosphate buffered saline (PBS).

### Primary cytotrophoblast isolation

As described previously [31, 32] approximately 150g of placental tissue was washed with sterile PBS and maternal and fetal surfaces were removed. Placental cotyledons were scraped with a scalpel to dissociate placental villi from vessels. Placental tissue was washed with 0.9% NaCl to remove blood cells then subjected to three 20 minute digestion cycles with 0.25% trypsin and 0.2mg/ml DNAse in Enzyme Digestion Buffer containing 10 x Hanks Buffered Salt Solution, sodium bicarbonate, HEPES and deionised H_2_O. Cell suspensions were filtered and then separated using a discontinuous Percoll gradient centrifugation. The layer containing cytotrophoblasts were then collected and subjected to a CD9 negative selection step, to remove contaminating non-trophoblast cells (resulting in >98% pure trophoblast population[33]). Primary cytotrophoblasts were plated at 5x10^5^/cm^2^ and cultured in DMEM high Glutamax (Thermofisher; Scoresby VIC) containing 10% Fetal Calf Serum (Sigma, St Louis, United States) and 1% anti-anti (Life Technologies) on fibronectin (10ug/mL; BD Bioscience, USA) coated plates, cells were cultured at 37°C under 8% O2. Viable cells attached overnight and were then washed twice with sterile PBS to remove non-viable cells and cell debris. Isolated primary cytotrophoblasts were treated with increasing doses of melatonin (1–1000μM) for 48 h. This culture period and cell density would include a mixed population of cytotrophoblast and syncytiotrophoblast [33].

### Isolation and culture of placental explants

Small pieces of villous tissue were cut from the mid-portion of the placenta to avoid the maternal and fetal surfaces. These were thoroughly washed with PBS and allowed to equilibrate at 37°C for 1 hour in DMEM (Thermofisher) containing 1% anti-anti and 10% fetal calf serum (Thermofisher). Tissue explants were then dissected into small fragments of 1-2mm size and three pieces put into each well of a 24 well plate and were cultured at 37°C under 8% O2. Placental explants were treated with melatonin (100μM and 1000μM) for 48h.

### Primary human umbilical vein endothelial cell (HUVEC) isolation

The cord vein of umbilical cords of normal term placentas was cannulated and infused with PBS to wash out fetal blood. Next, approximately 10ml (1mg/ml) of collagenase (Worthington, Lakewood, New Jersey) was infused into the cord followed by incubation at 37°C for 10 minutes. The dissociated HUVEC cells were recovered by pelleting and resuspension followed by culture in M199 media (Life Technologies) containing 20% fetal calf serum, 1% anti-anti and 1% endothelial cell growth factor (Sigma) and 1% heparin. HUVECs were cultured at 37°C under 20% O2. Isolated primary HUVECs (between passage 2 and 4) were treated with increasing doses of melatonin (Sigma (M5250); 1–1000μM) for 48 h.

### Endothelial dysfunction rescue studies

Endothelial cells (HUVECs) were treated with TNF-α (Thermofisher; 10ng/mL) to induce dysfunction. Cells were then cultured in the presence of either TNF-α (10ng/ml) alone; with both TNF-α (10ng/ml) and melatonin (50μM and 100μM); or control media for 8–12 h.

### Quantitative RT-PCR

Total RNA was extracted from isolated cytotrophoblast, endothelial cells (HUVEC) and placental explant tissue using the RNeasy mini kit (Qiagen, Valencia, CA) and quantified using a Nanodrop ND 1000 spectrophotometer (NanoDrop technologies Inc, Wilmington, DE) and converted to cDNA using Applied Biosystems high capacity cDNA reverse transcriptase kit (Thermofisher) as per manufacturer guidelines. Quantitative PCR was performed using Taqman gene expression assays for: HO-1, GCLC, TXN, NQO1, VCAM, ET-1. PCR was performed on the CFX 384 (Biorad, Hercules, CA) using FAM-labeled Taqman universal PCR mastermix (Applied Biosystems) with the following run conditions: 50 ^o^C for 2 minutes; 95 ^o^C for 10 minutes, 95 ^o^C for for 15 seconds, 60 ^o^C for 1 minute (40 cycles). All data were normalized to an appropriate house-keeping gene (isolated cells normalized to: GAPDH and YWHAZ; placental tissue to: TOP1 and CYC1) as an internal control and calibrated against the average C_t_ of the control samples. The results were expressed as fold change relative to controls. All samples were run in triplicate.

#### sFLT and sENG ELISAs

Conditioned media from primary cytotrophoblast, placental explants and endothelial cells (HUVECs) was assessed using ELISA for the presence of the following soluble factors: soluble Flt-1 (sFLT) DuoSet VEGF R1/Flt-1 kit (R&D systems by Bioscience, Waterloo, Australia), soluble endoglin (sENG) DuoSet Human Endoglin CD/105 (R&D systems). Optical density for all ELISAs was determined using a BioRad X-Mark microplate spectrophotometer (BioRad), Protein levels determined using BioRad Microplate manager 6 software. The assay lower detection limit was 125pg/mL and the upper detection limit was 8000pg/mL. The coefficients of variants were: intra-assay < 10% and inter-assay <15%.

#### Effects of melatonin on cell viability

Primary cytotrophoblasts and human umbilical vein endothelial cells (HUVECs) treated with increasing doses of melatonin (1–1000μM) showed no significant difference in cell viability (see S1 File). Cell viability assays were performed using CellTiter 96-Aqueous One solution (Promega, Madison WI) according to the manufacturer’s instructions.

### Statistical analysis

All *in vitro* and *ex vivo* experiments were performed with technical triplicates and all experiments were repeated a minimum of three times. Data was tested for normal distribution and statistically tested as appropriate. Data was tested non-parametrically using Kruskal-Wallis test. Data is expressed as mean ± SEM. P-values <0.05 were considered significant. Statistical analysis was performed using GraphPad Prism 6 software (GraphPad Software, La Jolla, CA).

## Results

### Effects of melatonin on anti-oxidant gene expression in primary human placental tissues and endothelial cells

**Fig 1:**
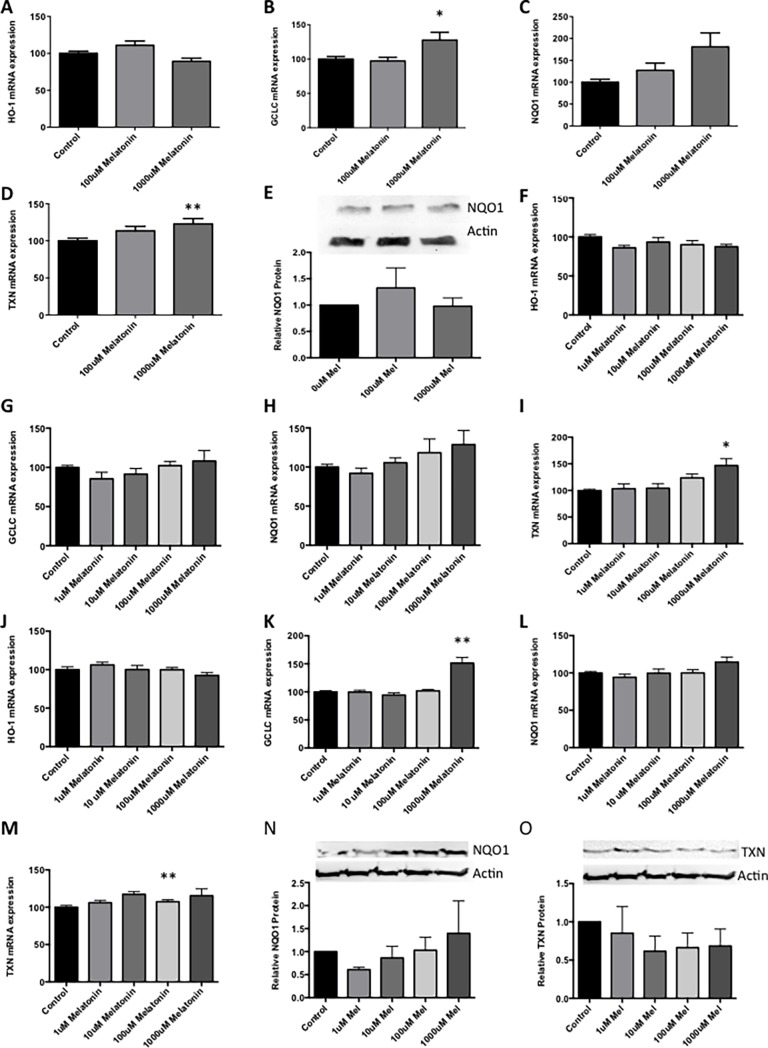
Effects of melatonin on anti-oxidant gene expression in primary human placenta. Normal term placental explant tissue, isolated primary cytotrophoblasts and human umbilical vein endothelial cells (HUVECs) were treated with melatonin (100–1000μM placental explant tissue and 1–1000μM for isolated cells) for 48 hours. Placental explant tissue mRNA expression for heme-oxygenase 1 (HO-1) (A), glutamate-cysteine ligase (GCLC) (B), NAD(P)H:quinone acceptor oxidoreductase 1 (NQO1) (C) and thioredoxin (TXN) (D) follwoing melatonin treatment. Densitometric analysis of placental explant NQO1 protein levels with melatonin treatment (E). Primary trophoblast mRNA expression of HO-1 (F), GCLC (G), NQO1 (H) and TXN (I) with melatonin treatment. Primary HUVEC mRNA expression of HO-1 (J), GCLC (K), NQO1 (L) and TXN (M). Experiments were performed with technical triplicates and all experiments were repeated a minimum of three times. Data is expressed as the mean % change from control ± SEM. All data were analyzed by Kruskal-Wallis followed by Dunn’s multiple comparisons test (*p≤ 0.05; **p≤ 0.01).

Melatonin was added to primary placental explants, and expression of anti-oxidant genes was measured. Melatonin had no effect on HO-1 mRNA expression (Fig 1A; see S2 File), but significantly increased expression of GCLC, NQO1 and TXN (Fig 1B–1D; see S2 File) at the top dose. However melatonin treatment did not increase NQO1 protein production by placental explants (Fig 1E; see S2 File).

When added to isolated primary trophoblast, melatonin treatment did not affect heme-oxygenase 1 (HO-1) expression (Fig 1F; see S2 File). Increasing doses of melatonin induced a non-significant trend toward an increase in mRNA expression of both glutamate-cysteine ligase (GCLC) and NAD(P)H:quinone acceptor oxidoreductase 1 (NQO1) (Fig 1G–1I). Melatonin significantly increased the mRNA expression of the anti-oxidant enzyme thioredoxin (TXN) (Fig 1I; see S2 File). When added to primary human umbilical vein endothelial cells (HUVECs), melatonin did not alter mRNA expression of HO-1 (Fig 1J; see S2 File), or NQO1 (Fig 1L; see S2 File), but significantly increased GCLC mRNA expression in HUVECs at the top dose (Fig 1K; see S2 File). Melatonin increased TXN mRNA expression at the 10μM concentration (Fig 1M; see S2 File). Consistently NQO1 protein was not increased in HUVECs with melatonin treatment, however while TXN mRNA was increased in HUVECs treated with melatonin (Fig 1M; see S2 File) there was no change in TXN protein in HUVECs treated with melatonin (Fig 1O; see S2 File).

In summary, addition of melatonin to primary placental tissues and cells caused an up-regulation in the mRNA expression of anti-oxidant genes. This possibly also occurs when administered to endothelial cells although the effects appear more modest.

### Effects of melatonin on sFLT and sENG secretion

The excess release of anti-angiogenic factors by the preeclamptic placenta into the maternal circulation is thought to lead to widespread maternal endothelial dysfunction and multi-organ injury seen in clinical disease. Drugs that can decrease the release of these factors may have potential as a treatment [31, 34, 35]. Therefore, we examined whether melatonin can decrease sFLT and sENG secretion from placental tissues and cells.

**Fig 2:**
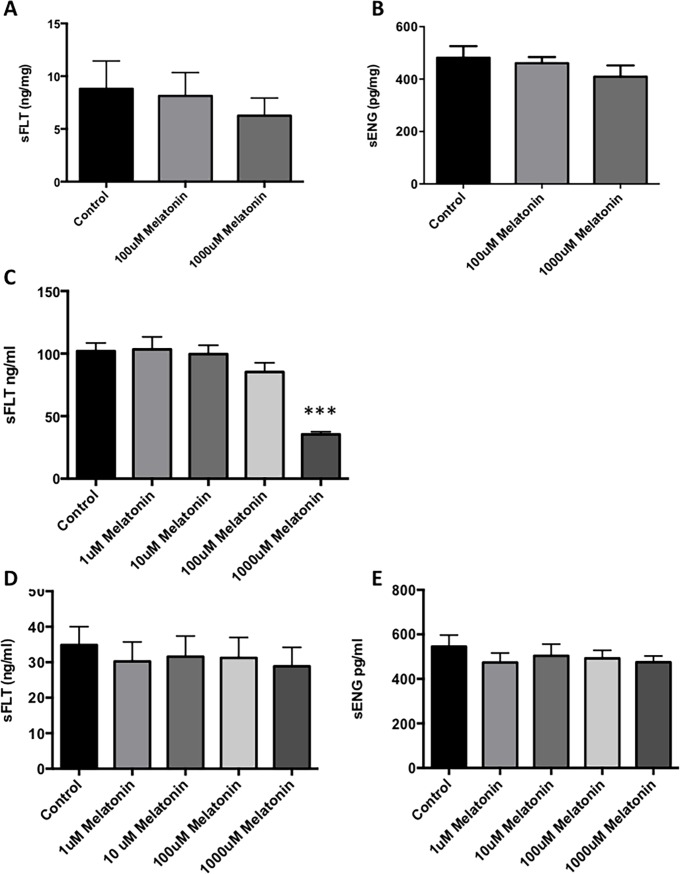
Effects of melatonin on sFLT and sENG secretion. Placental explant tissue, primary cytotrophoblasts and human umbilical vein endothelial cells (HUVECs) were treated with melatonin (100–1000μM placental explant tissue and 1–1000μM for isolated cells) for 48 h. Placental explant secretion of sFLT (A) and sENG (B) was assessed following melatonin treatment. Cytotrophoblast secretion of sFLT (C) following melatonin. HUVEC secretion of sFLT (D) and sENG (E) with increasing doses of melatonin. Experiments were performed with technical triplicates and all experiments were repeated a minimum of three times. Data is expressed as mean % change from control ± SEM. All data were analyzed by Kruskal-Wallis followed by Dunn’s multiple comparisons test (***p≤ 0.001).

Melatonin did not affect placental secretion of sFLT or sENG when administered to placental explants (Fig 2A and 2B; see S2 File). Melatonin significantly reduced sFLT secretion from primary human trophoblast at the 1000μM dose (Fig 2C; see S2 File). However, similarly to placental explant tissue, the addition of melatonin to HUVECs did not reduce either sFLT or sENG secretion (Fig 2D and 2E; see S2 File).

### Effects of melatonin on TNF-α induced endothelial dysfunction

**Fig 3:**
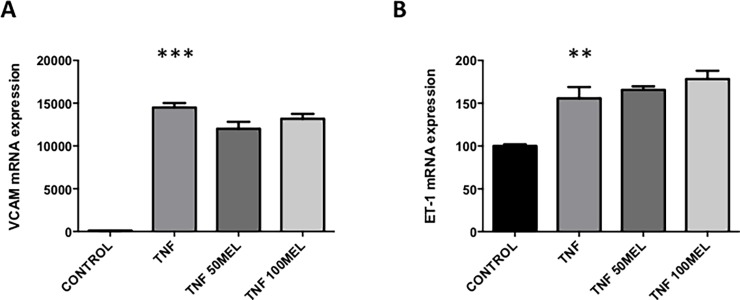
Effects of melatonin on TNF-α-induced endothelial dysfunction. Human umbilical vein endothelial cells (HUVECs) were treated with tumor necrosis factor α (TNF-α; 10ng/mL) for 2 hours, followed by addition of melatonin (50 and 100μM) in the presence of TNF-α (10 ng/mL) for an additional 24 hours. The mRNA expression of endothelial dysfunction markers was assessed VCAM (A) and ET-1 (B). Experiments were performed with technical triplicates and all experiments were repeated a minimum of three times. Data is expressed as relative mRNA expression ± SEM. All data were analyzed by Kruskal-Wallis followed by Dunn’s multiple comparisons test (**p≤ 0.01; ***p≤ 0.001).

Given endothelial dysfunction is an important aspect of the pathophysiology of preeclampsia, we examined whether melatonin might have actions to rescue endothelial dysfunction. As expected, administering tumor necrosis factor α (TNF-α) caused a potent increase in vascular cell adhesion molecule 1 (VCAM-1, Fig 3A; see S2 File) and Endothelin-1 (ET-1, Fig 3B; see S2 File), markers of endothelial dysfunction. The administration of melatonin at 50 and 100μM did not rescue TNF-α induced VCAM-1 or ET-1 mRNA up-regulation (Fig 3A and 3B; see S2 File).

In accordance with the PLOS ONE journal’s Data Availability requirements the full data sets are provided in a supporting information file S2 File.

## Discussion

Melatonin is a potent endogenous antioxidant hormone, with extrapineal synthesis occurring in the placenta throughout pregnancy. Both circulating melatonin and placental melatonin synthesis are decreased with preeclampsia, suggesting melatonin supplementation may be a potential therapeutic to treat the disease. Here we demonstrate for the first time that melatonin has increases antioxidant enzyme expression in placental tissue, with more modest effects on isolated trophoblast and endothelial cells (HUVECs). In addition, we show that melatonin has little to no effect on sFLT and sENG secretion, and does not mitigate TNF-α induced endothelial dysfunction in vitro.

In many disease models, melatonin has been shown to protect tissue from damage caused by inflammation and oxidative stress. Melatonin is a powerful antioxidant, acting as a direct scavenger of oxygen free radicals, especially the highly damaging hydroxyl radical, and indirectly through upregulation of antioxidant enzymes[36]. In animal models of fetal growth restriction (FGR), the administration of melatonin had positive effects on the newborn lamb with overall reduced hypoxia, improved neurodevelopment and decreased brain injury[37]. Furthermore in rodent models melatonin has been observed to have protective benefits in ischemic-reperfusion studies, reducing the overall induced oxidative damage to placental mitochondria[29], and melatonin administration was shown to improve the fetal to placental weight ratio, birth weight and enhance antioxidant enzyme production in a model of maternal undernourishment (which leads to oxidative damage) [38]. Given such promising data in animal models the PAMPR trial, an early Phase I (single arm, open label clinical trial) was established, to assess the potential clinical and biochemical effects of melatonin (daily oral administration (10 mg; 3 times)) in pregnancies complicated with pre-term preeclampsia [39].

Our group has a long standing interest in assessing drugs safe in pregnancy, to determine whether they may be able to mitigate the pathogenesis of preeclampsia. Our approach is to assess the efficacy of the agent to 1) enhance antioxidant pathways, 2) reduce the anti-angiogenic imbalance and 3) reduce maternal systemic oxidative stress and endothelial dysfunction. We have now shown that both metformin and the proton pump inhibitors are able to potently induce Nrf2 regulated anti-oxidant enzymes, reduce secretion of sFLT and sENG and mitigate endothelial dysfunction (in vitro and ex vivo)[40, 41] as well as hypertension in a mouse model of preeclampsia[41]. Thus using a dose range 1–1000μM of melatonin (previously shown to have actions on cytotrophoblast [42] and relevant to that being investigated in the PAMPR trial [39]) we aimed to assess whether melatonin was able to enhance antioxidant defences, reduce sFLT and sENG secretion and quench endothelial dysfunction. However we found that HO-1 production was not stimulated by melatonin in primary trophoblasts, placental explant tissue, or primary HUVECs.

In the current study, melatonin treatment upregulated the mRNA expression of ARE genes; TXN in primary trophoblasts, placental explant tissue and primary HUVECs; GCLC in placental explant tissue and primary HUVECs, but not primary trophoblasts; NQO1 dose-dependently in placental explant tissue, but not primary trophoblasts or primary HUVECs. However at these doses, melatonin was unable to increase the protein levels of these antioxidant molecules. Melatonin may act as an indirect antioxidant in trophoblast, placental explant tissue and primary HUVECs and may be useful to combat the rise in oxidative stress in the placenta during preeclampsia [43–45].

Anti-angiogenic factors (sFLT and sENG) are released in excess into the maternal circulation where they circulate causing widespread endothelial dysfunction [8, 9]. Quenching sFLT and sENG secretion would likely halt the damage to the maternal vasculature and end organ injury associated with preeclampsia. Melatonin treatment did not decrease the production of either sFLT or sENG by placental explant tissue or isolated primary HUVECs. However, the secretion of sFLT by primary trophoblast was significantly reduced following treatment with 1000 μM melatonin. It is however important to note, although not statistically significant, at this dose (1000 μM) melatonin had a slight negative effect on primary trophoblast cell viability, and as such this decrease in sFLT secretion may in fact reflect altered viability rather than a true protective effect of melatonin. In addition the placentae obtained and used in these experiments were collected from normal pregnancies. While the isolation of trophoblast and cell culture effects have been shown to enhance sFLT secretion[33] and thus provide a good model of preeclampsia in vitro, examination of Melatonin in placentae from preeclamptic pregnancies may provide further insight.

Our group [40, 41, 46–48] and others[49, 50] have used the pro-inflammatory cytokine TNF-α to model endothelial dysfunction in vitro with respect to preeclampsia. Importantly, endothelial sensitivity to TNF-α is enhanced by exposure to recombinant sFLT (as well as other agents that block VEGF signaling) [50]. We used TNF-α to induce endothelial dysfunction in vitro, consistent with our previous studies we found that TNF-α significantly increased expression of VCAM and ET-1. However melatonin was unable to mitigate the effects of TNF-α induced endothelial dysfunction in primary HUVECs. Interestingly, several animal models of vascular injury (smoke-induced and chronic intermittent hypoxia) have shown a recovery from endothelial dysfunction with melatonin treatment, reducing expression of both VCAM and ET-1 [51, 52]. Melatonin appears to have little ability to alleviate the effects of endothelial dysfunction in primary HUVECs isolated from the human placenta.

We have demonstrated that *in vitro*, melatonin (1–100μM) does not significantly quench sFLT and sENG production by placental explant tissue or primary HUVECs despite a decrease in sFLT production by primary trophoblasts (at top dose), nor does it mitigate the effects of endothelial dysfunction in primary HUVECs. Melatonin did however successfully act as an indirect antioxidant in these cells and tissues, differentially upregulating the expression of several ARE genes. It is still possible that melatonin’s antioxidant properties and safety profile may prove to be beneficial in maintaining an ongoing healthy pregnancy. We eagerly await the results of a phase 1 pilot clinical trial currently underway to test the effect of melatonin in preeclamptic pregnancies [39].

## Supporting information

S1 File Effects of melatonin on trophoblast and endothelial (HUVEC) viability. Primary cytotrophoblasts (A) and human umbilical vein endothelial cells (B) were isolated from term placentas and treated with increasing doses of melatonin (1–1000μM) for 48 h cell viability was assessed using a MTS assay. There was no significant effect on cell viability with melatonin treatment. Data is expressed as relative mRNA expression ± SEM. Data were analyzed by Kruskal-Wallis followed by Dunn’s multiple comparisons test.
(TIFF)

S2 File Raw data files and statistical analysis used for generation of Figs 1–3. (PDF)
