# Supplementary figures and images for: Melatonin enhances antioxidant molecules in the placenta, reduces secretion of soluble fms-like tyrosine kinase 1 (sFLT) from primary trophoblast but does not rescue endothelial dysfunction: An evaluation of its potential to treat preeclampsia
Source: PLoS One. 2018 Apr 11;13(4):e0187082. doi: 10.1371/journal.pone.0187082 (PMC5894956; doi:10.1371/journal.pone.0187082)

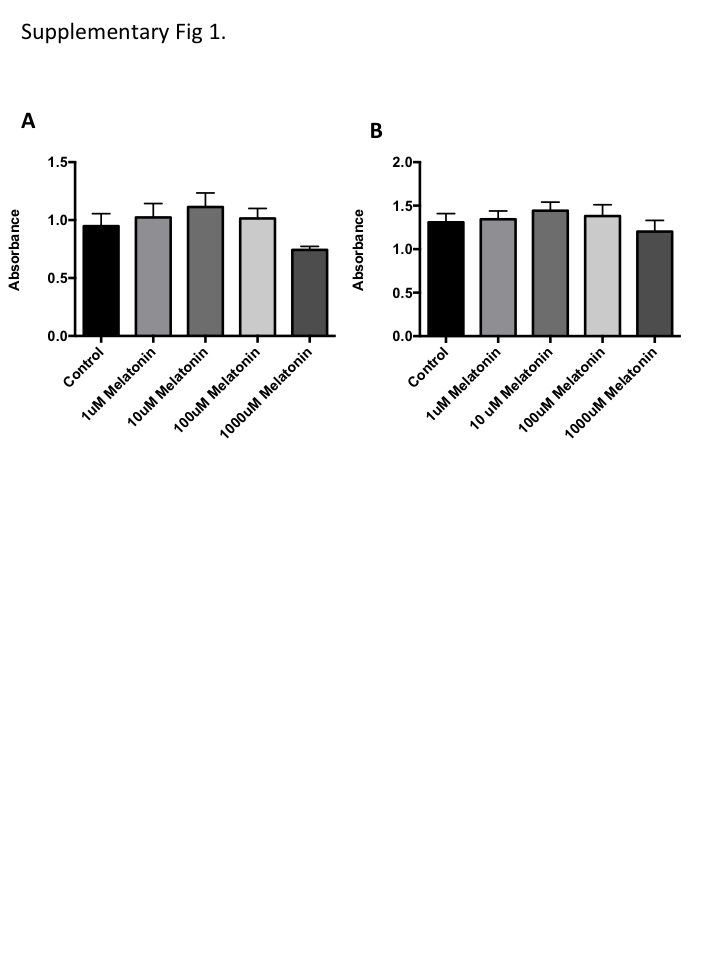

Supplement: S1 File — Primary cytotrophoblasts (A) and human umbilical vein endothelial cells (B) were isolated from term placentas and treated with increasing doses of melatonin (1–1000μM) for 48 h cell viability was assessed using a MTS assay. There was no significant effect on cell viability with melatonin treatment. Data is expressed as relative mRNA expression ± SEM. Data were analyzed by Kruskal-Wallis followed by Dunn’s multiple comparisons test. (TIFF) [file pone.0187082.s001.tiff]
