# Supplementary material for: Melatonin enhances antioxidant molecules in the placenta, reduces secretion of soluble fms-like tyrosine kinase 1 (sFLT) from primary trophoblast but does not rescue endothelial dysfunction: An evaluation of its potential to treat preeclampsia
Source: PLoS One. 2018 Apr 11;13(4):e0187082. doi: 10.1371/journal.pone.0187082 (PMC5894956; doi:10.1371/journal.pone.0187082)
Supplement: S2 File — (PDF) [file pone.0187082.s002.pdf]

Placental explant tissue HO-1 mRNA expression values (Figure 1A)

Control	100uM Melatonin	1000uM Melatonin
99.77	127.66	76.61
99.37	131.46	80.52
100.86	113.05	95.57
105.93	85.82	67.33
90.52	121.57	84.72
103.56	89.53	107.06
81.84	98.21	102.61
109.49	128.75	89.32
108.67	102.26	99.4

Kruskal Wallis test of HO-1 mRNA expression placental explant data (Fig 1A)

Number of families	1				
Number of comparisons per family	2				
Alpha	0.05				
Dunn's multiple comparisons test	Mean rank diff.	Significant?	Summary		
Control vs. 100uM Melatonin	-3.556	No	ns		
Control vs. 1000uM Melatonin	6.222	No	ns		
Test details	Mean rank 1	Mean rank 2	Mean rank d	n1	n2
Control vs. 100uM Melatonin	14.89	18.44	-3.556	9	9
Control vs. 1000uM Melatonin	14.89	8.667	6.222	9	9

Placental explant tissue GCLC mRNA expression values (Figure 1B)

Control	100uM Melatonin	1000uM Melatonin
98.12	88.04	125.54
103.86	112.51	132.76
98.01	91.83	105.07
103.91	77.94	110.61
86.03	98.58	126.62
110.05	75.11	109.58
80.26	101.61	214.5
103.49	123.94	117.51
116.26	105.62	105.86

Kruskal Wallis test of GCLC mRNA expression placental explant data (Fig 1B)

Number of families	1				
Number of comparisons per family	2				
Alpha	0.05				
Dunn's multiple comparisons test	Mean rank diff.	Significant?	Summary		
Control vs. 100uM Melatonin	0.6667	No	ns		
Control vs. 1000uM Melatonin	-10.33	Yes	*		
Test details	Mean rank 1	Mean rank 2	Mean rank d	n1	n2
Control vs. 100uM Melatonin	10.78	10.11	0.6667	9	9
Control vs. 1000uM Melatonin	10.78	21.11	-10.33	9	9

Placental explant tissue NQO1 mRNA expression values (Figure 1C)

Control	100uM Melatonin	1000uM Melatonin
126.76	128.63	
91.15	111.75	133.59
82.09	244.28	76.43
104.94	78.05	169.07
80.5	111.9	128.6
114.57	73.25	123.36
81.32	140.1	241.82
131.77	137.55	210.98
86.91	118.96	363.05

Kruskal Wallis test of NQO1 mRNA expression placental explant data (Fig 1C)

Number of families	1			
Number of comparisons per family	2			
Alpha	0.05			
Dunn's multiple comparisons test	Mean rank d	Significant?	Summary	
Control vs. 100uM Melatonin	-4.111	No	ns	
Control vs. 1000uM Melatonin	-8.917	Yes	*	
Test details	Mean rank 1	Mean rank 2	Mean rank d	n1
Control vs. 100uM Melatonin	9.333	13.44	-4.111	9
Control vs. 1000uM Melatonin	9.333	18.25	-8.917	9

Placental explant tissue TXN mRNA expression values (Figure 1D)

Control	100uM Melatonin	1000uM Melatonin
110.01	99.14	112.47
94.94	119.64	106.65
95.05	153.41	92.54
116.63	101.08	127.69
86.44	121.82	118.41
96.93	99.01	123.88
91.71	106.99	124
114.58	122.31	126.7
93.71	97.68	172.48

Kruskal Wallis test of TXN mRNA expression placental explant data (Fig 1D)

Number of families	1				
Number of comparisons per family	2				
Alpha	0.05				
Dunn's multiple comparisons test	Mean rank diff.	Significant?	Summary		
Control vs. 100uM Melatonin	-7.222	No	ns		
Control vs. 1000uM Melatonin	-10.78	Yes	**		
Test details	Mean rank 1	Mean rank 2	Mean rank d	n1	n2
Control vs. 100uM Melatonin	8	15.22	-7.222	9	9
Control vs. 1000uM Melatonin	8	18.78	-10.78	9	9

Densitometric values of placental explant protein treated with Melatonin (100 -100uM) Figure 1E (n=4)

**Samples**	**Control**	**100uM Melatonin**	**1000uM Melatonin**
N412	1	0.934	0.720
N413	1	2.462	1.127
N414	1	1.015	0.710
N415	1	0.895	1.357

Kruskal-Wallis test of densitometric values of placental explant NQO1 WB Figure 1E (n=4)

Number of families	1				
Number of comparisons per family	2				
Alpha	0.05				
Dunn's multiple comparisons test	Mean rank d	Significant?	Summary		
0uM Mel vs. 100uM Mel	-0.5	No	ns		
0uM Mel vs. 1000uM Mel	0.5	No	ns		
Test details	Mean rank 1	Mean rank 2	Mean rank d	n1	n2
0uM Mel vs. 100uM Mel	6.5	7	-0.5	4	4
0uM Mel vs. 1000uM Mel	6.5	6	0.5	4	4

Trophoblast HO-1 mRNA expression values (Figure 1F)

Sample	Control	1uM Melatonin	10uM Melatonin	100uM Melatonin	1000uM Melatonin
N117A	90.78	98.94	84.24	97.59	98.04
N117B	90	78.18	80.41	76.69	76.56
N117C	119.22	76.36	75.18	68.07	84.27
N131A	100	75.92	92.53	92.33	79.06
N131B	96.87	77.22	75.76	98.53	96.35
N131C	103.12	99.6	89.63	92.99	97.26
N134A	96.12	88.88	110.94	117.09	91.32
N134B	109.85	87.96	107.99	69.71	73.58
N134C	94.04	91.25	124.31	97.52	90.65

Kruskal Wallis test of HO-1 mRNA expression Trophoblast data (Fig 1F)

Number of families	1				
Number of comparisons per family	4				
Alpha	0.05				
Dunn's multiple comparisons test	Mean rank d	Significant?	Summary		
Control vs. 1uM Melatonin	13.89	No	ns		
Control vs. 10uM Melatonin	9	No	ns		
Control vs. 100uM Melatonin	9.111	No	ns		
Control vs. 1000uM Melatonin	12.44	No	ns		
Test details	Mean rank 1	Mean rank 2	Mean rank d	n1	n2
Control vs. 1uM Melatonin	31.89	18	13.89	9	9
Control vs. 10uM Melatonin	31.89	22.89	9	9	9
Control vs. 100uM Melatonin	31.89	22.78	9.111	9	9
Control vs. 1000uM Melatonin	31.89	19.44	12.44	9	9

Trophoblast GCLC mRNA expression values (Figure 1G)

Samples	Control	1uM Melatonin	10uM Melatonin	100uM Melatonin	1000uM Melatonin
N117A	103.15	77.97	89.13	95.32	88.24
N117B	95.56	77.85	68.72	83.88	85.71
N117C	101.28	62.09	64.69	86.43	146.69
N131A	93.84	125.8	133.61	99.35	120.96
N131B	97.84	105.71	106.13	124.45	172.16
N131C	108.32	120.25	109.83		136.25
N134A	111.83	69.15	84.96	97.75	50.11
N134B	101.66	66.55	79.08	105.86	58.06
N134C	86.51	63.96	86.82	124.63	113.91

Kruskal Wallis test of GCLC mRNA expression Trophoblast data (Fig 1G)

Number of families	1				
Number of comparisons per family	4				
Alpha	0.05				
Dunn's multiple comparisons test	Mean rank diff.	Significant?	Summary		
Control vs. 1uM Melatonin	9.333	No	ns		
Control vs. 10uM Melatonin	5.778	No	ns		
Control vs. 100uM Melatonin	0.06944	No	ns		
Control vs. 1000uM Melatonin	-0.7778	No	ns		
Test details	Mean rank 1	Mean rank 2	Mean rank d	n1	n2
Control vs. 1uM Melatonin	25.44	16.11	9.333	9	9
Control vs. 10uM Melatonin	25.44	19.67	5.778	9	9
Control vs. 100uM Melatonin	25.44	25.38	0.06944	9	8
Control vs. 1000uM Melatonin	25.44	26.22	-0.7778	9	9

Trophoblast NQO1 mRNA expression values (Figure 1H)

Sample	Control	1uM Melatonin	10uM Melatonin	100uM Melatonin	1000uM Melatonin
N117A	98.72	92.01	89.99	83.92	100.78
N117B	80.06	64.23	87.64	95.58	96.91
N117C	121.23	79.78	76.06	91.83	81.69
N131A	94.89	99.7	109.18	120.91	185.01
N131B	105.31	127.48	121.66	115.67	225.36
N131C	99.8	115.58	136.28	255.09	186.6
N134A	100.26	95.58	101.78	96.17	82.07
N134B	100.13	75.85	110.71	89.36	93.83
N134C	99.61	77.38	116.89	115.76	106.17

Kruskal Wallis test of NQO1 mRNA expression Trophoblast data (Fig 1H)

Number of families	1				
Number of comparisons per family	4				
Alpha	0.05				
Dunn's multiple comparisons test	Mean rank d	Significant?	Summary		
Control vs. 1uM Melatonin	7.5	No	ns		
Control vs. 10uM Melatonin	-2.778	No	ns		
Control vs. 100uM Melatonin	-1.5	No	ns		
Control vs. 1000uM Melatonin	-3.222	No	ns		
Test details	Mean rank 1	Mean rank 2	Mean rank d	n1	n2
Control vs. 1uM Melatonin	23	15.5	7.5	9	9
Control vs. 10uM Melatonin	23	25.78	-2.778	9	9
Control vs. 100uM Melatonin	23	24.5	-1.5	9	9
Control vs. 1000uM Melatonin	23	26.22	-3.222	9	9

Trophoblast TXN mRNA expression values (Figure 1I)

Sample	Control	1uM Melatonin	10uM Melatonin	100uM Melatonin	1000uM Melatonin
N117A	110.01	89.24	70.48	124.82	118.06
N117B	98.75	87.24	82.35	102.5	96.75
N117C	91.24	68.05	72.45	101.44	163.21
N131A	86.65	132.2	140.78	125.01	148.33
N131B	104.4	128.73	121.06	138.41	205.42
N131C	108.95	151.89	129.36	173.12	199.45
N134A	99.3	98.84	86.84	124.51	109.37
N134B	103.11	85.53	115.02	107.37	128.03
N134C	97.58	89.04	119.38	114.97	152.81

Kruskal Wallis test of TXN mRNA expression Trophoblast data (Fig 1I)

Number of families	1				
Number of comparisons per family	4				
Alpha	0.05				
Dunn's multiple comparisons test	Mean rank d	Significant?	Summary		
Control vs. 1uM Melatonin	-1.444	No	ns		
Control vs. 10uM Melatonin	-3	No	ns		
Control vs. 100uM Melatonin	-12.11	No	ns		
Control vs. 1000uM Melatonin	-17.89	Yes	*		
Test details	Mean rank 1	Mean rank 2	Mean rank d	n1	n2
Control vs. 1uM Melatonin	16.11	17.56	-1.444	9	9
Control vs. 10uM Melatonin	16.11	19.11	-3	9	9
Control vs. 100uM Melatonin	16.11	28.22	-12.11	9	9
Control vs. 1000uM Melatonin	16.11	34	-17.89	9	9

HUVEC HO-1 mRNA expression values (Figure 1J)

Sample	Control	1uM Melatonin	10 uM Melatonin	100uM Melatonin	1000uM Melatonin
N127A	76.47	116.76	76.78	88.29	89.64
N127B	108.44	82.98	81.61	97.51	80.31
N127C	115.09	98.48	107.21	91.34	107.98
N128A	94.67	111.09	90.49	93.26	72.02
N128B	96.03	111.22	126	100.81	94.37
N128C	109.3	118.46	111.49	105.41	89.96
N129A	95.29	107.61	95.22	99.07	102.3
N129B	105.01	100.58	101.91	106.57	100.48
N129C	99.7	108.47	110.84	116.76	95.74

One Way ANOVA of HO-1mRNA exprssion HUVEC (Fig 1J)

Number of families	1
Number of comparisons per family	4
Alpha	0.05

Dunnett's multiple comparisons test	Mean Diff.	95% CI of diff.	Significant?	Summary
Control vs. 1uM Melatonin	-6.183	-20.32 to 7.957	No	ns
Control vs. 10 uM Melatonin	-0.1722	-14.31 to 13.97	No	ns
Control vs. 100uM Melatonin	0.1089	-14.03 to 14.25	No	ns
Control vs. 1000uM Melatonin	7.467	-6.673 to 21.61	No	ns

Test details	Mean 1	Mean 2	Mean Diff.	SE of diff.	n1	n2	q	DF
Control vs. 1uM Melatonin	100	106.2	-6.183	5.56	9	9	1.112	40
Control vs. 10 uM Melatonin	100	100.2	-0.1722	5.56	9	9	0.03097	40
Control vs. 100uM Melatonin	100	99.89	0.1089	5.56	9	9	0.01958	40
Control vs. 1000uM Melatonin	100	92.53	7.467	5.56	9	9	1.343	40

HUVEC GCLC mRNA expression values (Figure 1K)

Sample	Control	1uM Melatonin	10 uM Melatonin	100uM Melatonin	1000uM Melatonin
N127A	89.87	110.61	78.41	107.9	155.04
N127B	103.82	102.85	86.54		140.19
N127C	106.31	102.56	108.57	108.37	136.12
N128A	92.47	108.97	78.52	94.74	165.64
N128B	97.31	99.04	96.56	97.49	194.8
N128C	110.21	99	112.35	103.84	200.1
N129A	98.38	78.94	90.22	96.03	119.95
N129B	106.45	87.21	101.72	96.94	124.18
N129C	95.17	106.91	97.33	110.25	124.47

Kruskal-Wallis test of GCLC mRNA expression HUVEC (Fig 1K)

Number of families	1				
Number of comparisons per family	4				
Alpha	0.05				
Dunn's multiple comparisons test	Mean rank diff.	Significant?	Summary		
Control vs. 1uM Melatonin	-2.111	No	ns		
Control vs. 10 uM Melatonin	3.889	No	ns		
Control vs. 100uM Melatonin	-2.486	No	ns		
Control vs. 1000uM Melatonin	-22.11	Yes	**		
Test details	Mean rank 1	Mean rank 2	Mean rank d	n1	n2
Control vs. 1uM Melatonin	17.89	20	-2.111	9	9
Control vs. 10 uM Melatonin	17.89	14	3.889	9	9
Control vs. 100uM Melatonin	17.89	20.38	-2.486	9	8
Control vs. 1000uM Melatonin	17.89	40	-22.11	9	9

HUVEC NQO1 mRNA expression values (Figure 1L)

Sample	Control	1uM Melatonin	10 uM Melatonin	100uM Melatonin	1000uM Melatonin
N127A	97.56	94.36	93.61	78.91	98.77
N127B	98	88.62	78.74	101.35	97.1
N127C	104.44	79.19		85.38	81.02
N128A	91.34	105.93	109.94	110.5	125.79
N128B	97.99	96.34	128.08	114.25	145.77
N128C	110.68	113.21	110.07	98.93	135.34
N129A	96.87	78.57	97.94	100.73	121.53
N129B	96.22	85.59	93.2	92.8	104.7
N129C	106.91	106.14	86.4	117.49	120.48

Kruskal-Wallis test of NQO1 mRNA expression HUVEC (Fig 1L)

Number of families	1				
Number of comparisons per family	4				
Alpha	0.05				
Dunn's multiple comparisons test	Mean rank diff.	Significant?	Summary		
Control vs. 1uM Melatonin	5.667	No	ns		
Control vs. 10 uM Melatonin	1.639	No	ns		
Control vs. 100uM Melatonin	-0.7778	No	ns		
Control vs. 1000uM Melatonin	-9.333	No	ns		
Test details	Mean rank 1	Mean rank 2	Mean rank d	n1	n2
Control vs. 1uM Melatonin	21.89	16.22	5.667	9	9
Control vs. 10 uM Melatonin	21.89	20.25	1.639	9	8
Control vs. 100uM Melatonin	21.89	22.67	-0.7778	9	9
Control vs. 1000uM Melatonin	21.89	31.22	-9.333	9	9

HUVEC TXN mRNA expression values (Figure 1M)

Sample	Control	1uM Melatonin	10uM Melatonin	100uM Melatonin	1000uM Melatonin
N127A	96.88	112.72	106.75	123.2	
N127B	103.41	97.07	122.99	107.43	95.2
N127C	99.71	101.68	109.59	111.94	98.08
N128A	101.15	111.86	111.67	95.95	126.26
N128B	93.7	116.12	135.9	104.47	166.25
N128C	105.16	121.76	132.47	102.32	134.72
N129A	96.57	98.69	103.34	102.5	109.05
N129B	114.1	92.78	116.13	106.91	85.85
N129C	89.33	101.59	116.66	112.02	108.48

Kruskal-Wallis test of TXN mRNA expression HUVEC (Fig 1M)

Number of families 1

Number of comparisons per family	4				
Alpha	0.05				
Dunn's multiple comparisons test	Mean rank diff.	Significant?	Summary		
Control vs. 1uM Melatonin	-7.222	No	ns		
Control vs. 10uM Melatonin	-18.78	Yes	**		
Control vs. 100uM Melatonin	-9.667	No	ns		
Control vs. 1000uM Melatonin	-10.9	No	ns		
Test details	Mean rank 1	Mean rank 2	Mean rank d	n1	n2
Control vs. 1uM Melatonin	13.22	20.44	-7.222	9	9
Control vs. 10uM Melatonin	13.22	32	-18.78	9	9
Control vs. 100uM Melatonin	13.22	22.89	-9.667	9	9
Control vs. 1000uM Melatonin	13.22	24.13	-10.9	9	8

Densitometric values of NQO1 protein levels from HUVEC treated with Melatonin (1 - 1000uM) Figure 1N (n=4)

**Samples**	**Control**	**1uM Melatonin**	**10uM Melatonin**	**100uM Melatonin**	**1000uM Melatonin**
N397	1	0.4690417	0.4673041	0.4590714	0.8322385
N398	1	0.5872875	0.7550396	0.9735136	0.5303297
N406	1	0.6556988	1.60412	1.817362	3.498247
N409	1	0.7228286	0.6242386	0.8713989	0.73011

Kruskal-Wallis test of NQO1 protein levels from HUVEC treated with Melatonin (1 - 1000uM) Figure 1N (n=4)

Number of families	1				
Number of comparisons per family	4				
Alpha	0.05				
Dunn's multiple comparisons test	Mean rank diff.	Significant?	Summary		
Control vs. 1uM Mel	9.75	No	ns		
Control vs. 10uM Mel	6.5	No	ns		
Control vs. 100uM Mel	4.25	No	ns		
Control vs. 1000uM Mel	4.5	No	ns		
Test details	Mean rank 1	Mean rank 2	Mean rank d	n1	n2
Control vs. 1uM Mel	15.5	5.75	9.75	4	4
Control vs. 10uM Mel	15.5	9	6.5	4	4
Control vs. 100uM Mel	15.5	11.25	4.25	4	4
Control vs. 1000uM Mel	15.5	11	4.5	4	4

Densitometric values of TXN protein levels from HUVEC treated with Melatonin (1 - 1000uM) Figure 1O (n=4)

**Samples**	**Control**	**1uM Melatonir**	**10uM Melatonir**	**100uM Melaton**	**1000uM Melatonin**
N396	1	0.2827663	0.1416651	0.1896097	0.1633708
N397	1	0.5025878	0.5796622	0.5147341	0.7248393
N406	1	0.7737096	0.62523	1.01358	0.6050533
N409	1	1.850595	1.116108	0.9321194	1.244827

Kruskal-Wallis test of TXN protein levels from HUVEC treated with Melatonin (1 - 1000uM) Figure 1O (n=4)

Number of families	1				
Number of comparisons per family	4				
Alpha	0.05				
Dunn's multiple comparisons test	Mean rank diff	Significant?	Summary		
Control vs. 1uM Mel	4.5	No	ns		
Control vs. 10uM Mel	5.75	No	ns		
Control vs. 100uM Mel	5	No	ns		
Control vs. 1000uM Mel	4.75	No	ns		
Test details	Mean rank 1	Mean rank 2	Mean rank d	n1	n2
Control vs. 1uM Mel	14.5	10	4.5	4	4
Control vs. 10uM Mel	14.5	8.75	5.75	4	4
Control vs. 100uM Mel	14.5	9.5	5	4	4
Control vs. 1000uM Mel	14.5	9.75	4.75	4	4

sFlt secretion from placental explants (Fig 2A)

**Sample**	**Control**	**100uM Melatonin**	**1000uM Melatonin**
N144A	4961.685	4266.375	2587.64
N144B	3218.826	2816.472	2251.805
N144C	2924.173	2703.3	1989.318
N145A	3969.348	4634.213	3355.74
N145B	4065.244	3801.98	5225.41
N145C	5541.884	4673.98	2515.68
N157A	12473.58	18574.17	13638.93
N157B	16173.61	13404.63	11684.37
N1457C	25942.09	18261.57	13071.8

Kruskal Wallis test of sFlt1secretion by placental explant (Fig 2A)

Number of families	1				
Number of comparisons per family	2				
Alpha	0.05				
Dunn's multiple comparisons test	Mean rank diff.	Significant?	Summary		
Control vs. 100uM Melatonin	0.7778	No	ns		
Control vs. 1000uM Melatonin	4.889	No	ns		
Test details	Mean rank 1	Mean rank 2	Mean rank d	n1	n2
Control vs. 100uM Melatonin	15.89	15.11	0.7778	9	9
Control vs. 1000uM Melatonin	15.89	11	4.889	9	9

sENG secretion from placental explants (Fig 2B)

**Sample**	**Control**	**100uM Melatonin**	**1000uM Melatonin**
N144A	518.401	483.7105	421.7575
N144B	368.3474	359.3362	373.878
N144C	386.986	498.1376	304.4748
N145A	398.6288	345.6007	312.418
N145B	376.4684	435.834	290.4837
N145C	445.3704	490.98	392.35
N157A	772.4134	561.79	673.818
N157B	589.106	462.0896	350.9868
N1457C	481.8812	504.4874	561.9593

Kruskal Wallis test of sENG secretion by placental explant (Fig 2B)

Number of families	1				
Number of comparisons per family	2				
Alpha	0.05				
Dunn's multiple comparisons test	Mean rank diff.	Significant?	Summary		
Control vs. 100uM Melatonin	0.3333	No	ns		
Control vs. 1000uM Melatonin	5.667	No	ns		
Test details	Mean rank 1	Mean rank 2	Mean rank d	n1	n2
Control vs. 100uM Melatonin	16	15.67	0.3333	9	9
Control vs. 1000uM Melatonin	16	10.33	5.667	9	9

sFlt secretion from trophoblast (Fig 2C)

**Sample**	**Control**	**1uM Melatonin**	**10uM Melatonin**	**100uM Melatonin**	**1000uM Melatonin**
N117A	89.75708	75.808	79.62839	66.87601	29.76984
N117B	77.33121	78.49032	80.70826	70.89299	27.63826
N117C	80.38297	74.44467	77.61088	68.51316	37.61243
N131A	131.1541	149.0274	115.5363	107.0409	45.85166
N131B	122.2656	146.587	119.2526	116.0273	42.2609
N131C	124.2966	127.3066	129.8952	119.3928	36.13908
N134A	99.90068	87.82294	81.92659	74.20667	31.68226
N134B	100.2778	101.3712	95.26092	77.12186	39.3375
N134C	93.29843	89.97595	117.866	68.06797	28.11375

Kruskal Wallis test of sFlt HUVEC data (Fig 2C)

Number of families	1				
Number of comparisons per family	4				
Alpha	0.05				
Dunn's multiple comparisons test	Mean rank diff.	Significant?	Summary		
Control vs. 1uM Melatonin	1.333	No	ns		
Control vs. 10uM Melatonin	1.333	No	ns		
Control vs. 100uM Melatonin	10.44	No	ns		
Control vs. 1000uM Melatonin	25.78	Yes	***		
Test details	Mean rank 1	Mean rank 2	Mean rank d	n1	n2
Control vs. 1uM Melatonin	30.78	29.44	1.333	9	9
Control vs. 10uM Melatonin	30.78	29.44	1.333	9	9
Control vs. 100uM Melatonin	30.78	20.33	10.44	9	9
Control vs. 1000uM Melatonin	30.78	5	25.78	9	9

sFlt secretion from HUVEC (Fig 2D)

Sample	Control	1uM Melatonin	10 uM Melatonin	100uM Melatonin	1000uM Melatonin
N127A	46483.83	46284.42	45217.86	46740.37	41923.4
N127B	45885.9	41459.19	45047.49	40336.36	41346.74
N127C	45374.11	44196.73	46997.06	45203.66	41754.52
N128A	46214.89	39645.5	39161.32	41071.65	37171.43
N128B	42778.31	38617.45	41497.56	41269.33	36180.99
N128C	44140.09	36255.91	40631.05	41528	38119.67
N129A	13651.23	8358.952	8149.568	8687.168	7533.504
N129B	14939.16	8746.744	8314.12	8344.008	8014.72
N129C	14383.56	8627.56	9029.376	7804.544	7804.544

Kruskal Wallis test of sFlt HUVEC data (Fig 2D)

Number of families	1				
Number of comparisons per family	4				
Alpha	0.05				
Dunn's multiple comparisons test	Mean rank d	Significant?	Summary		
Control vs. 1uM Melatonin	8.889	No	ns		
Control vs. 10 uM Melatonin	6.222	No	ns		
Control vs. 100uM Melatonin	7.5	No	ns		
Control vs. 1000uM Melatonin	13.5	No	ns		
Test details	Mean rank 1	Mean rank 2	Mean rank d	n1	n2
Control vs. 1uM Melatonin	30.22	21.33	8.889	9	9
Control vs. 10 uM Melatonin	30.22	24	6.222	9	9
Control vs. 100uM Melatonin	30.22	22.72	7.5	9	9
Control vs. 1000uM Melatonin	30.22	16.72	13.5	9	9

sENG secretion from HUVEC (Fig 2E)

**Sample**	**Control**	**1uM Melatonin**	**10uM Melatonin**	**100uM Melatonin**	**1000uM Melatonin**
N127A	531.257	427.724	406.165	492.23	487.29
N127B	454.963	377.066	410.332	431.208	447.965
N127C	443.771	411.027	424.938	423.545	447.266
N128A	398.382	405.571	365.668	420.759	396.231
N128B	408.454	364.259	403.411	379.11	379.82
N128C	434.608	362.851	389.792	392.651	384.087
N129A	788.17	674.692	694.382	631.896	580.474
N129B	680.056	592.858	766.42	626.562	562.812
N129C	762.802	649.7	672.904	631.896	587.548

Kruskal Wallis test of sENG HUVEC data (Fig 2E)

Number of families	1				
Number of comparisons per family	4				
Alpha	0.05				
Dunn's multiple comparisons test	Mean rank diff.	Significant?	Summary		
Control vs. 1uM Melatonin	9.889	No	ns		
Control vs. 10uM Melatonin	6.778	No	ns		
Control vs. 100uM Melatonin	5.444	No	ns		
Control vs. 1000uM Melatonin	6.778	No	ns		
Test details	Mean rank 1	Mean rank 2	Mean rank d	n1	n2
Control vs. 1uM Melatonin	28.78	18.89	9.889	9	9
Control vs. 10uM Melatonin	28.78	22	6.778	9	9
Control vs. 100uM Melatonin	28.78	23.33	5.444	9	9
Control vs. 1000uM Melatonin	28.78	22	6.778	9	9

Endothelial dysfunction; VCAM mRNA expression (HUVECs treated with TNFa) Figure 3A (n=3)

Samples	CONTROL	TNFa	TNFa + 50uM Melatonin	TNFa + 100uM Melatonin
N10A	84.01	20715.14	17536.46	18508.59
N10B	117.36	14397.46	14851.26	18526.86
N10C	98.63	17424.07	15581.27	15028.63
N12A	95.48	2316.51	3274.14	3031.41
N12B	101.30	2767.05	3168.50	3659.10
N12C	103.22	2704.85	3104.36	2715.32
N13A	96.51	23204.17	19679.83	15495.86
N13B	100.09	23568.34	17460.55	20647.54
N13C	103.40	23363.09	13443.99	20998.18

Kruskal Wallis test of VCAM mRNA expression in endothelial dysfunction assay (n=3) Figure 3A

Number of families	1				
Number of comparisons per family	3				
Alpha	0.05				
Dunn's multiple comparisons test	Mean rank d	Significant?	Summary		
TNF vs. CONTROL	19	Yes	***		
TNF vs. TNF 50MEL	2.333	No	ns		
TNF vs. TNF 100MEL	0.6667	No	ns		
Test details	Mean rank 1	Mean rank 2	Mean rank d	n1	n2
TNF vs. CONTROL	24	5	19	9	9
TNF vs. TNF 50MEL	24	21.67	2.333	9	9
TNF vs. TNF 100MEL	24	23.33	0.6667	9	9

Endothelial dysfunction; ET-1 mRNA expression (HUVECs treated with TNFa) Figure 3B (n=3)

**Samples**	**CONTROL**	**TNFa**	**TNFa + 50uM Melatonin**	**TNFa + 100uM Melatonin**
N10A	102.07	179.86	177.38	197.89
N10B	105.03	131.58	158.27	173.71
N10C	92.9	167.16	161.71	163.12
N12A	104.66	97.3	170.4	144.58
N12B	105.68	128.18	145.31	171.09
N12C	89.67	109.32	154.95	138.47
N13A	106.85	198.85	171.67	181.99
N13B	93.15	189.58	185.81	204.34
N13C	100.01	199.99	164.82	229.3

Kruskal Wallis test of ET-1 mRNA expression in endothelial dysfunction assay (n=3) Figure 3B

Number of families	1				
Number of comparisons per family	3				
Alpha	0.05				
Dunn's multiple comparisons test	Mean rank d	Significant?	Summary		
TNF vs. CONTROL	15.22	Yes	**		
TNF vs. TNF 50MEL	-0.8889	No	ns		
TNF vs. TNF 100MEL	-4.778	No	ns		
Test details	Mean rank 1	Mean rank 2	Mean rank d	n1	n2
TNF vs. CONTROL	20.89	5.667	15.22	9	9
TNF vs. TNF 50MEL	20.89	21.78	-0.8889	9	9
TNF vs. TNF 100MEL	20.89	25.67	-4.778	9	9
